# Resonant Ultrasound Spectroscopy Detection Using a Non-Contact Ultrasound Microphone

**DOI:** 10.3390/s25196154

**Published:** 2025-10-04

**Authors:** Jake Pretula, Nolan Shaw, Ayden Chen, Kyle G. Scheuer, Ray G. DeCorby

**Affiliations:** 1 Department of Electrical and Computer Engineering, University of Alberta, 9211-116 St. NW, Edmonton, AB T6G 1H9, Canada; jpretula@ualberta.ca (J.P.); nshaw@ualberta.ca (N.S.); 2 Ultracoustics Technologies Ltd., 10230 Jasper Ave. NW, Edmonton, AB T5J 4P6, Canada; ayden@ultracoustics.ca (A.C.); kyle@ultracoustics.ca (K.G.S.)

**Keywords:** resonant ultrasound spectroscopy, optomechanical sensors, non-destructive testing, air-coupled ultrasound, ultrasonic inspection, ultrasound microphone

## Abstract

We observed vibrational eigenmodes for a variety of millimeter-scale objects, including glass and sapphire lenses, by placing them on a piezoelectric ‘shaker’ driven by a broadband noise or frequency sweep signal, and using an optomechanical microphone to pick up their vibrational signatures emitted into the surrounding air. High-quality vibrational modes were detected over the ~0–8 MHz range for a typical object–microphone spacing of 1–10 mm. The observed eigenfrequencies are shown to be in excellent agreement with numerical predictions. Non-contact detection of resonant vibrational eigenmodes in the MHz ultrasound range could find application in the quality control of numerous industrial parts, such as ball bearings and lenses.

## 1. Introduction

In resonant ultrasound spectroscopy (RUS) [1,2,3,4,5], the vibrational modes of a small, solid object are measured in order to ascertain the elastic constants of a material or for assessing the quality (i.e., size, shape, and presence of defects) in manufactured parts [6]. RUS is typically applied to objects with dimensions in the mm to cm range, yielding low-order vibrational resonances in the kHz to MHz range. The most common RUS setup employs a pair of contact-mode piezoelectric transducers (i.e., one for excitation and one for detection) with the object of interest held ‘lightly’ [1,3] in-between and treated as having approximately free boundary conditions.

These conventional setups are complex and present challenges with respect to repeatability. For example, very small contact forces are needed in order to minimally perturb the ‘free’ boundary conditions of the sample and to avoid damaging delicate samples. This leads to relatively weak transduction of the acoustic signals and thus a need for sophisticated electronics and signal processing techniques. A typical system uses a frequency sweep (step) [3] excitation combined with synchronous (i.e., lock-in) detection, and a broadband scan can take up to several minutes to complete [4,7]. Moreover, in order to capture all of the vibrational modes in a frequency range of interest, contact-mode setups often require multiple measurement runs, with the object mounted in a different orientation for each measurement [3,4]. While not a major deterrent for studies of elastic constants, these features are not ideally suited to the rapid inspection and quality control (QC) of manufactured objects. Non-contacting approaches, particularly if sensitive enough to enable rapid data collection, might expand the application space for RUS techniques.

The earliest demonstration of RUS [8] employed a single shear piezo to both excite and detect torsional modes of a sphere, which was simply rested atop the transducer. Non-contact detection methods for ‘resting’ spheres were subsequently reported, for example using electromagnetic–acoustic transduction (EMAT) with metallic spheres [9] or laser interferometric readout of ceramic balls [10]. As an extension of the latter work, Yamanaka et al. [11] floated steel balls on pressurized air columns and performed RUS measurements by using a combination of pulsed laser excitation and laser interferometric detection. Electrical microphones or air-coupled piezoelectric transducers have occasionally been used for non-contact detection in RUS setups [12], but are typically limited to frequencies below ~100 kHz. Here, we show that an ultra-sensitive optomechanical microphone [13,14,15] can enable non-contact and rapid sensing of mechanical resonances excited by a low-voltage, broadband noise source in the ~0–8 MHz range. High SNR datasets, revealing the main vibrational modes of various objects, were obtained on sub-second time-scales, and compare favorably to data collected using a slower approach combining frequency sweep and synchronous detection. The described system might find applications for in-line, non-destructive testing (NDT) and quality control (QC) of small industrial parts such as lenses, bearings [6], or additively manufactured objects [16].

## 2. Materials and Methods

**Figure 1:**
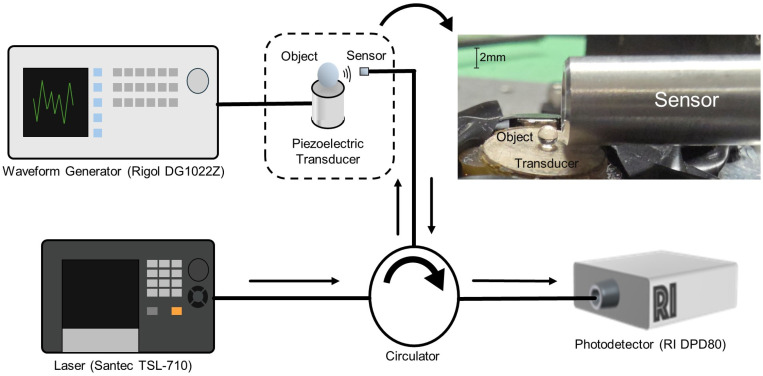
Overall schematic of the experimental setup, with an inset photograph of the experimental RUS assembly featuring the optomechanical ultrasound microphone, an object of interest (2 mm N-BK7 ball lens shown in callout), and the piezoelectric transducer used to excite vibrational modes in the object.

The experimental setup is shown in Figure 1. Objects of interest were placed on a conventional (i.e., contact-mode) piezoelectric transducer driven by a low-cost function generator. The transducer excites vibrational modes of the object, which in turn radiates acoustic waves into the surrounding air, and these waves are detected using an optomechanical microphone. The microphone is similar to those described elsewhere [13,14,15] and is essentially a fiber-coupled Fabry–Perot cavity with a flexible upper mirror acting as a mechanical oscillator. The cavity itself has nearly perfect cylindrical symmetry and thus negligible polarization dependence, and the entire setup employs standard (i.e., non-polarization-maintaining) fibers and components. The sensors operate in a thermo-mechanical-noise-limited regime [17] and can thus enable broadband detection of acoustic signals extending from the low kHz range up to the high MHz range. In fact, for operation in air, it is the rapid scaling of ultrasound attenuation with frequency [18] that imposes the practical upper limit on the bandwidth. The microphone used here has its first fundamental resonance at ~3.9 MHz and a noise-equivalent-pressure (NEP) on the order of 50 μPa Hz^−1/2^ [13]. Since the main goal of the present manuscript is to describe a non-contact RUS technique, a detailed description of the microphone and its calibration is left for the Supplementary Materials Document.

The microphone readout employs a laser, tuned to the side of a fundamental optical resonance of the cavity (i.e., the ‘tuned to slope’ technique), and the motions of the flexible cavity mirror (i.e., due to both intrinsic thermal vibrations and external acoustic pressure waves) are imprinted on the reflected laser light. The reflected light is delivered to a high-speed photodetector (DPD80, Resolved Instruments Inc., Edmonton, Canada) with a bandwidth of 40 MHz, and the acoustic response is extracted from the time-varying received optical power. Data collection employed Resolved Instruments software (PteroDAQtyl 1.1.0), and the data was subsequently processed using MATLAB to produce the frequency-domain plots shown below. Additional details on the detector settings and subsequent signal processing are described in the Supplementary Materials Document.

Detection of the vibrational modes for an object of interest was accomplished by resting the object on a ‘source’ piezoelectric transducer. To enhance acoustic coupling to the object, a small water droplet was first placed on the transducer surface using a pin or a sharp pair of tweezers, and the object was then rested on top of the droplet. Excess water was found to dampen the vibrational modes of the object and produce unwanted spectral content associated with the vibrational modes of the droplet itself [19], so that it was often necessary to let most of the water evaporate (tens of seconds typically) before high-quality data could be collected. Development of a more repeatable and reliable technique for object-to-transducer coupling would be necessary in an industrial setting. For example, soft gel pads are often employed in dry-coupled ultrasound tank level sensors, and it might be possible to adapt such an approach to our RUS setup. Exploration of these details is left for future work.

For most of the results below, the transducer was driven by a waveform generator (DG1022Z, Rigol Technologies Inc., Portland, OR, USA) producing a broadband (0–25 MHz), 20 V_P-P_ noise signal. The broadband noise signal has a larger bandwidth than the ‘source’ transducers used. For example, we mainly employed a non-resonant transducer with peak response at ~4 MHz, and non-negligible response over a bandwidth of approximately 0–8 MHz (see the Supplementary Materials File for additional details). The transducer bandwidth and the air attenuation were thus the main factors limiting the range over which the eigen-frequencies of an object could be observed. An object excited by the broadband noise signal vibrates preferentially at its mechanical eigen-frequencies, emitting ultrasonic waves into its surrounding environment to be detected by the optomechanical ultrasound microphone. Due to the aforementioned extreme attenuation of ultrasound signals in air at MHz-range frequencies, it was necessary to limit the separation between the object and the optomechanical microphone to less than ~10 mm. To maximize the SNR of high-frequency modes, most of the results below were obtained with an object–microphone spacing on the order of 1–3 mm. By driving the transducer with a noise source, a full spectrum of vibrational modes could be extracted in less than 1 s. Furthermore, in most cases the SNR was sufficient to reveal nearly all of the predicted vibrational resonances, as evidenced by the results below. Nevertheless, significantly higher SNR measurements could be made using the same setup but driving the transducer in a frequency sweep (i.e., stepping the frequency of a sinusoidal drive) mode combined with synchronous detection using a software-based lock-in technique [11]. The higher SNR comes at the cost of greatly increased measurement time (typically several minutes).

## 3. Results

Using the setup described above, we studied a variety of glass, quartz, sapphire, and steel objects. Experimental spectra were compared to theoretical results obtained using both a numerical solver (COMSOL) and using an on-line analytical solver [20]. For simplicity, objects were assumed to have ‘free’ boundary conditions, which neglects the (typically small) impact of the piezoelectric transducer on which they are rested [21]. Nevertheless, very good agreement between the observed and predicted resonant vibrational modes was found in all cases. Representative results follow; additional experimental data and a more complete description of the theoretical techniques and results can be found in the Supplementary Materials File.

### 3.1. Spherical Ball Lenses

We begin with results for commercially available, 2 mm-diameter ball lenses, one composed of N-BK7 glass (#32-744, Edmund Optics Inc., Barrington, IL, USA) and a second composed of sapphire crystal (Edmund Optics #43-642, Edmund Optics Inc., Barrington, IL, USA). RUS of spherical objects, which are uniquely amenable to analytical solution of their vibrational eigen-modes, is sometimes called the ‘resonant sphere technique’ (RST) [1]. An elastic sphere hosts both purely torsional and so-called ‘spheroidal’ vibrational modes, the latter comprising both torsional and radial displacements of the sphere [22,23]. We used conventional piezoelectric transducers with predominately out-of-plane displacement, which are not expected to efficiently couple the torsional modes of a resting sphere. Moreover, since torsional modes do not involve displacement of the sphere surface, they do not directly excite acoustic waves in the surrounding air. Consistent with these facts, our experiments revealed only the spheroidal modes. These modes are often labeled as *S*_mn_, where *m* indicates the order of a spherical Bessel function (associated with orbital angular momentum), and *n* indicates the number of nodes in the radial direction [22]. For example, the *S*_0n_ modes are purely radial breathing modes of the sphere. The frequency ordering of the modes depends on Poisson’s ratio, but the lowest frequency spheroidal mode is always the *S*_21_ eigen-mode [7,22]. We labeled the calculated eigen-frequencies according to this convention; additional details including simulated mode-field patterns are provided in the Supplementary Materials File.

**Figure 2:**
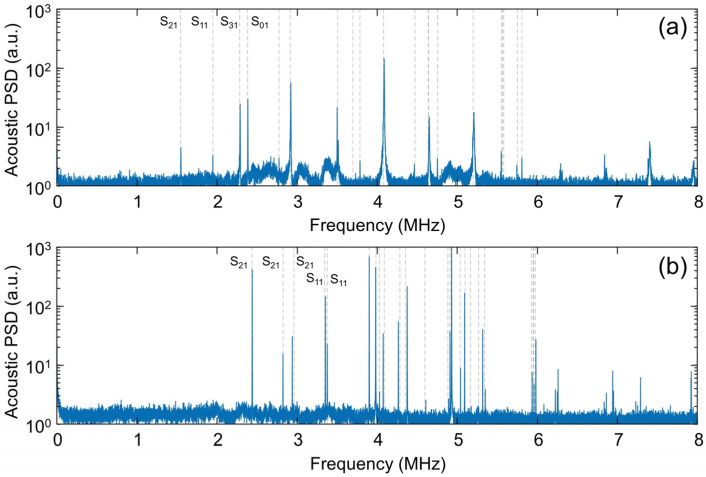
Acoustic power spectral density (PSD) for the 2 mm N-BK7 ball lens (**a**) and 2mm sapphire ball lens (**b**). The vertical dashed lines indicate the theoretically predicted eigen-frequencies for the spheroidal modes, calculated using an analytical solver. For the sapphire lens, anisotropy has been included in the model (see Supplementary Materials File).

Typical plots of the acoustic power spectral density received by the optomechanical microphone are shown in Figure 2. This data was collected by placing either the N-BK7 or sapphire ball lens on the transducer and positioning the microphone within ~2 mm of the lens. The low-amplitude ripple in these datasets is due to acoustic energy coupled directly into the air by the piezoelectric transducer, as verified by collecting spectra with no object in place (see the Supplementary Materials File). Of greater interest here is the collection of high-Q peaks, which we attribute to the resonant vibrational eigen-modes of the spherical lens. This is supported by the excellent alignment between these peaks and the theoretically predicted (spheroidal) eigen-frequencies calculated using an online analytical solver [20] and overlaid as gray dotted lines in these plots. These eigen-mode predictions were also confirmed using COMSOL. For the models, we used elastic constants reported in the literature and summarized in the Supplementary Materials File. For nominally isotropic N-BK7 glass, scalar values for elastic modulus, density, and Poisson’s coefficient were used and taken from the built-in COMSOL library [24], while for anisotropic sapphire, the elastic constants are described in terms of a 6 × 6 tensor [25]. Good agreement between the experimental and predicted eigen-frequencies was achieved over the entire ~0–6 MHz range. While a few vibrational modes were typically detected above 6 MHz, they are only a subset of the predicted spheroidal modes in this high-frequency range. This can be attributed to the low response of the excitation transducer at these frequencies, combined with rapidly scaling attenuation of acoustic signals in air. As shown below, it was possible to reveal additional modes by driving the transducer with a sinusoidal input and employing phase-sensitive detection, but at the expense of increased measurement times.

For both lenses, the predicted spheroidal eigen-frequencies are globally well aligned with the sharp peaks in the experimental spectrum. The manufacturer states a tolerance of ~0.1% on the ball lens diameters, and we have verified that dimensional uncertainty is not the main contributor to the residual discrepancies between theory and experiment (i.e., the slightly mis-aligned resonance frequencies, which are visible for some of the higher-order modes). A more likely reason is that the elastic constants assumed in the models are only approximately correct for these lenses. In any case, since our main goal here is to demonstrate and validate the non-contact RUS detection technique, and not to conduct a detailed study of elastic material properties, we have not attempted to place error bars on these plots. The lower amplitude of some of the experimental peaks can be attributed to lower excitation by the transducer, or to lower coupling of some vibrational modes into acoustic waves captured by the microphone. In fact, we typically found that the relative amplitude of the modes was dependent on the particular position of the microphone with respect to the object. As per the discussion above, purely torsional modes of the spheres were not experimentally observed.

It is worth emphasizing that the experiment captured all of the predicted spheroidal modes below ~6 MHz, even for the highly anisotropic sapphire ball. For sapphire, anisotropy results in ‘splitting’ of nominally degenerate modes (i.e., modes sharing the same mode indices *m* and *n*), with the modal eigen-frequency greatly dependent on the mode-field pattern relative to the crystallographic axes. For example, the three lowest-order modes observed in Figure 2b at ~2.4, 2.8, and 3 MHz are all *S*_21_ modes, each oriented along its own orthogonal direction in three-dimensional space. Signatures of each were captured in a single measurement, with the microphone position fixed and the transducer driven by wideband noise, as detailed above. More information regarding the numerically predicted modes and their mode-field images is available in the Supplementary Materials Document.

### 3.2. Splitting of Nominally Degenerate Modes Attributable to Residual Anisotropy of 1 mm N-BK7 Lens

**Figure 3:**
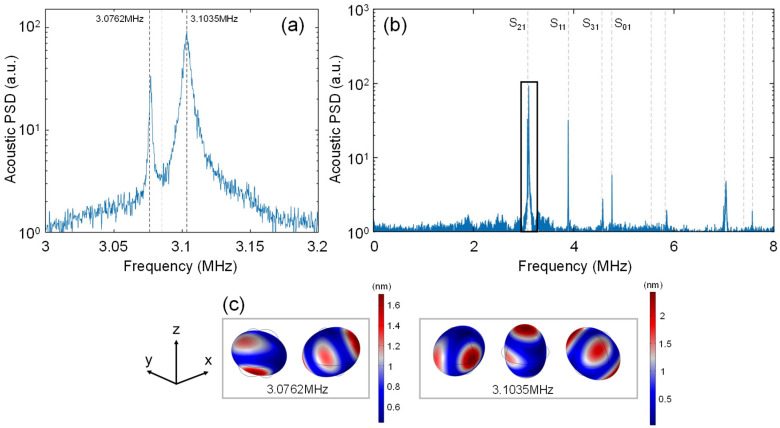
Plots showing the mode splitting of the fundamental eigen-frequency of a 1 mm N-BK7 ball lens. (**a**) Zoom-in of the fundamental mode of the 1mm ball lens; the light gray line represents the numerically predicted eigen-frequency of the perfectly isotropic ball lens, whereas the black dotted lines represent the numerically predicted eigen-frequencies with slight anisotropy introduced into the COMSOL model. (**b**) Expanded spectrum shown over the 0–8 MHz range. The box indicates the region plotted in part (**a**). (**c**) COMSOL-predicted mode-field images for a slightly anisotropic ball and associated with the eigen-frequencies, indicated by the black dotted lines in part (**a**).

As discussed above, RUS can be used for the QC of small manufactured parts. For example, our RUS setup was able to discern signatures of residual anisotropy and/or asphericity for the commercial N-BK7 lenses studied. A representative result is shown in Figure 3 for a 1 mm N-BK7 ball lens (Edmund Optics #43-708), which exhibited ‘splitting’ of its fundamental mode, predicted near ~3.1 MHz, into two adjacent lines separated by ~350 kHz.

Note that the fundamental *S*_21_ mode of the perfectly symmetric, isotropic sphere is actually 5-fold degenerate [26]. Slight eccentricity or anisotropy can lift this degeneracy. The manufacturer’s specified asphericity for this lens (<0.625 μm [27]) is not sufficiently large to produce the magnitude of the splitting observed. We therefore speculate that the splitting is mainly due to a residual anisotropy of the glass, which would split the *S*_21_ mode into two ‘branches’ [28], consistent with the experimental data. In fact, it is known that the stresses introduced during glass formation or subsequent steps can induce slight anisotropy of a nominally amorphous glass [29]. To assess this possibility, we used COMSOL to simulate the eigen-modes in the presence of slight anisotropy, employing a simple ‘cubic’ anisotropy model (i.e., three independent values in the elasticity tensor) for the N-BK7 glass. To fit the predictions of the anisotropic model to the experimental data, the elasticity tensor of N-BK7 glass was freely and slightly adjusted around the starting values for the perfectly isotropic material, and within a range consistent with reported anisotropies for manufactured glass objects [29]. In Figure 3a, the numerically predicted ‘split’ eigen-frequencies for a spherical but slightly anisotropic lens are indicated by the black dotted lines, while the light gray line represents the degenerate eigen-frequency for a perfectly isotropic and spherical lens. The predicted mode-field patterns for the two branches of split eigen-modes are shown in Figure 3c. More information on the COMSOL simulation and the precise values used for the elasticity tensor are provided in the Supplementary Materials Document.

The anisotropy-induced-splitting explanation is admittedly speculative, since we were not able to verify the glass anisotropy by other means. In any case, the experimental results further demonstrate the potential of our technique for the QC of lenses and other manufactured objects. In particular, the noise-based RUS technique enables a spectrum to be captured in a few hundred milliseconds, suggesting possibilities for rapid, in-line inspection of parts.

### 3.3. Fused Silica Half-Ball Lens—Comparison of Noise Source and Frequency Sweep Techniques

**Figure 4:**
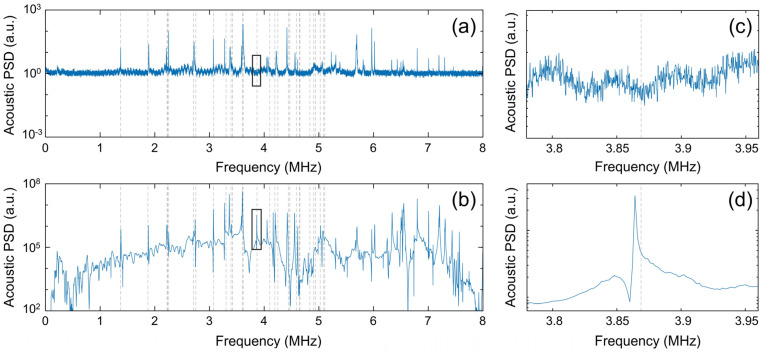
(**a**) Received acoustic PSD for the 1.5 mm fused silica half-ball lens, obtained using the noise-excitation-based RUS technique. (**b**) The acoustic PSD for the same object, but recorded using a frequency sweep excitation and synchronous detection technique. The boxes indicate the regions plotted in parts (**c**) and (**d**), respectively. The Fano-resonance mode at ~3.87 MHz, and several others at higher frequency, were only revealed using the frequency sweep technique.

As a final example, this section shows representative results for a non-spherical object and furthermore compares results obtained using the noise-excitation method to those obtained using a slower, sinusoidal-sweep and synchronous detection method. The non-spherical object considered is a 1.5 mm diameter fused silica half-ball lens, which was placed with its flat side facing upwards to minimize its contact with the piezoelectric transducer. The vibrational eigen-modes were recorded using the rapid, noise-based RUS methodology described above (see Figure 4a) and also using a slower technique, which combined a frequency sweep (peak-to-peak amplitude of 3 V) with a software-based lock-in detection method [11] (see Figure 4b). In each of these plots, the predicted eigen-frequencies obtained using COMSOL are indicated by the vertical dashed lines. We used a simple isotropic model for the fused silica (from the built-in COMSOL library [24]), which produced good agreement between experiment and theory over at least the ~0–5 MHz range.

For comparison purposes, both datasets were plotted over a six orders of magnitude range on the vertical axis. However, note the sinusoidal excitation results in the received acoustic PSD lying ~5 orders of magnitude higher than for the noise-based excitation. In spite of this, the SNR (~10–1000) of the main vibrational features of the object is quite similar in the two cases. This is because the noise floor in the sinusoidal excitation case is dominated by the background acoustic energy emitted into the air by the transducer, and this noise floor increases in proportion to the increased energy of the object’s vibrational modes. This also reveals features of the transducer below ~1 MHz, which are of low acoustic intensity (below ~10^5^ a.u.) and which we attribute to the non-flat response of the transducer in this range. For the noise-based excitation, the acoustic radiation from the transducer is only weakly evident (see also Figure 1 and Figure 2), and the noise floor is mainly set by thermo-mechanical and shot noise sources inherent to the optomechanical microphone.

Nevertheless, the higher signal level for the frequency sweep method does enable additional features in the acoustic spectra to be revealed. For example, Figure 4c,d show that the sweep method revealed a theoretically predicted resonant mode near ~ 3.87 MHz, which was ‘missed’ by the noise-excitation scan. From inspection of Figure 4a,b, it is clear that the frequency sweep technique revealed several additional modes, especially in the frequency range above ~5 MHz, where the transducer response is weak. Another interesting property of the sweep-based measurement is that many of the observed resonant lines have an obvious ‘Fano-resonance’ [30] characteristic. We believe this arises from the coupling between the low-Q vibrational modes of the source transducer and the higher-Q modes of the object of interest. It might be interesting to explore ways to suppress the background acoustic energy radiated by the piezo transducer, in order to better isolate the vibrational modes of the object of interest. Possibilities include reducing the area of the transducer so that it is mostly covered by the object, or using some means to selectively attenuate the path between the transducer surface and the microphone. Exploration of these ideas is left for future work.

## 4. Discussion and Conclusions

In summary, we have demonstrated air-coupled detection of the vibrational eigen-modes for various millimeter-scale objects rested on a piezoelectric transducer, using an optomechanical microphone. Nearly complete sets of (radiating) vibrational eigen-modes, spanning several MHz in bandwidth, could be extracted in sub-second measurement times by driving the exciting transducer with a low-voltage noise signal from a function generator. This unique RUS approach is largely enabled by the sensitive, broadband, and omnidirectional response of the optomechanical microphone employed [13,14,15]. It has advantages over conventional RUS setups, which are subject to repeatability issues (related to variability in contact forces and dependence on object mounting and orientation) and which typically require a slower measurement scheme combining frequency-sweep excitation with synchronous detection. It could also augment or complement systems designed for non-contact RUS characterization of plates [31]. Air attenuation sets the practical upper limit on the bandwidth of our technique, and thus also sets a lower limit on the smallest objects that could be inspected. For example, the 1 mm diameter lens studied has its lowest resonance near ~3 MHz, and since we can detect features up to ~8 MHz, objects on the order of hundreds of microns are likely representative of the minimum object size that could be studied in practice.

Our RUS technique could be well suited to the assembly-line QC of manufactured parts. Beyond the simple ball and half-ball lenses used to demonstrate the technique here, there is potential to apply the technique to more complex objects such as nuts, screws, etc. by using numerical approaches to predict their vibrational eigen-frequencies. A set of eigen-frequencies can be used to assess the size and shape of a manufactured object, given a set of assumed elastic material properties. On the other hand, material properties can be assessed by assuming the size and shape. As an example, we showed that residual anisotropy in N-BK7 glass could be extracted from close analysis of the RUS results for a spherical ball lens. Additional examples of measurements on small steel objects (bearing balls and dowel pins) are provided in the Supplementary Materials. Generally speaking, it is possible to extract details encompassing size, shape, and elastic material constants from a single RUS dataset but using a sufficiently sophisticated inversion and fitting procedure [4,5]. Rapid extraction of a vibrational spectrum as described here, combined with machine learning and AI algorithms, might allow the geometry and material properties of an object to be determined on sub-second timescales. These are interesting avenues for further exploration, but are left for future work.

## Data Availability

The data that support the findings of this study are available from the authors upon reasonable request.

## Supplementary Materials

The following supporting information can be downloaded at https://www.mdpi.com/article/10.3390/s25196154/s1: Figure S1: Schematic of the experimental setup from the main manuscript. Figure S2: Acoustic spectrum of the piezoelectric transducer without an object lying atop it, also displaying normalization methods. Table S1: Table displaying the material properties and simulation method used for each of the objects. Figure S3: Mode-field images for the spheroidal modes of a 2 mm N-BK7 and 2 mm sapphire ball lens. Figure S4: Experimental acoustic spectra of a small ball bearing and small steel dowel pin. References [13,14,20,24,25,32] are cited in the Supplementary Materials.

## Abbreviations

The following abbreviations are used in this manuscript:

RUS	Resonant Ultrasound Spectroscopy
NEP	Noise equivalent pressure
PSD	Power spectral density
DSP	Digital signal processing
