# Supplementary material for: Resonant Ultrasound Spectroscopy Detection Using a Non-Contact Ultrasound Microphone
Source: Sensors (Basel). 2025 Oct 4;25(19):6154. doi: 10.3390/s25196154 (PMC12526948; doi:10.3390/s25196154)
Supplement: Supplementary file 1 [file sensors-25-06154-s001.zip › sensors-3783358-supplementary.pdf]

Supplemental Information for "Resonant Ultrasound Spectroscopy Detection using a Non-contact Ultrasound Microphone"

J. Pretula,[1] N. Shaw,[1] A. Chen,[2] K. G. Scheuer,[2] and R. G. DeCorby [1,2,*]

[1] ECE Department, University of Alberta, 9211-116 St. NW, Edmonton, AB, Canada, T6G 1H9;
[2] Ultracoustics Technologies Ltd., 10230 Jasper Ave. NW, Edmonton, AB, Canada T5J 4P6
* Correspondence: rdecorby@ualberta.ca (R.G.D)

1. Additional experimental details

1.1. *Experimental setup*

For ease of reference in the discussion below, the experimental schematic from the main manuscript is reproduced in Fig. S1. A tunable laser is delivered to the optomechanical microphone (*i.e.*, labeled as the sensor in the schematic diagram), and its wavelength is adjusted to be slightly detuned from an optical resonance of the sensor. In this way, variations in the optical cavity length due to thermal vibrations or incident pressure waves (*i.e.*, ultrasound) are mapped to intensity variations in the light delivered to a high-speed photodetector receiver. For all of the measurements described in the present work, the average received power at the photodetector was ~100 µW. The RI photoreceiver digitizes received signals at 80 MSamples/sec, and digitized waveforms were saved to a computer and subsequently processed in MATLAB. The optomechanical sensors used have been described in much greater detail in our previous publications [1-3]. Nevertheless, a few pertinent details for the specific sensor used here are provided below.

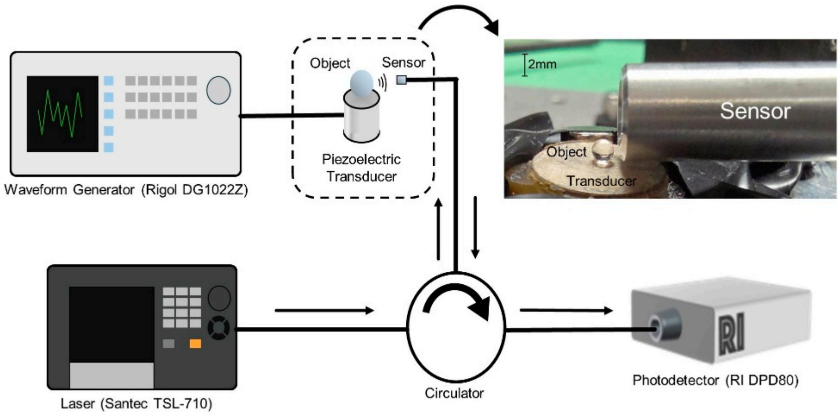

Figure S1. Schematic diagram of the experimental setup, reproduced from the main manuscript. Objects of interest are rested on a piezoelectric transducer driven by a function generator. Vibrational eigen-modes of the object that couple to acoustic propagating waves in the air are detected using an optomechanical microphone positioned within a few mm of the object.

1.2. *The optomechanical sensor and detection of incident acoustic pressure waves*

The optomechanical sensor is a 'buckled dome' microcavity [1], in which a flexible upper mirror acts as a mechanical oscillator. Thermal energy and external forces induce motion of this mirror, which in turn modulates the length of an optical cavity. An interrogation laser tuned to the slope of an optical resonance maps changes in cavity length to changes in the intensity of light reflected by the cavity. The buckled dome sensor is essentially a half-symmetric, spherical mirror Fabry-Perot cavity [1] and supports high-quality Laguerre-Gaussian (LG) modes in the 1550 nm-wavelength range. The laser is tuned near a fundamental LG mode with a $Q > 1000$ and linewidth of < 1 nm. The sensor of interest was aligned and coupled to an SMF-28 fiber cable, with all parts permanently fixed in a microphone assembly [2,3].

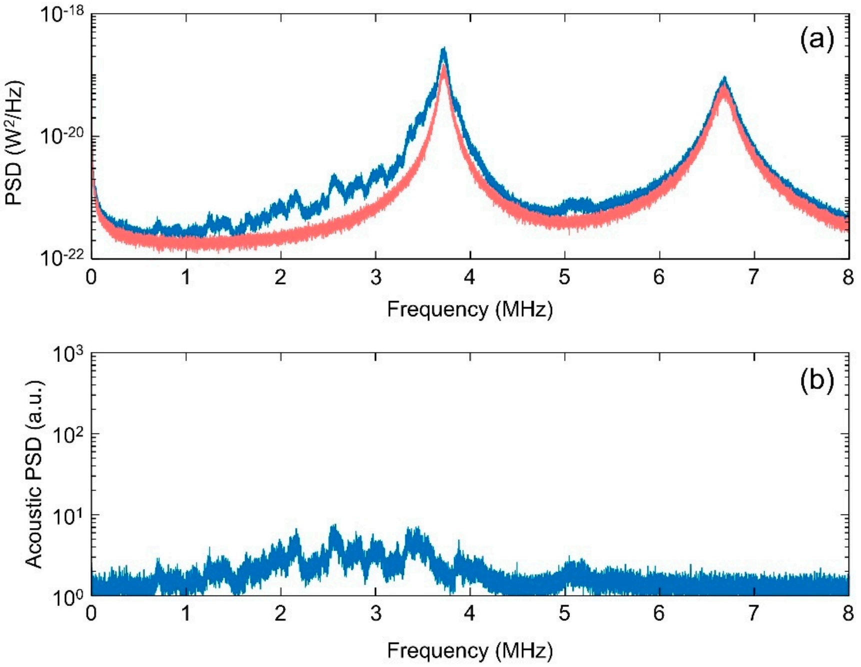

Figure S2. (a) Spectrum recorded by the optomechanical sensor without (red) and with (blue) a broadband noise source driving the piezoelectric transducer. For these measurements, the microphone was positioned a few mm above the transducer, and no additional object was present. (b) The normalized spectrum for the noise-driven transducer. This curve was obtained by dividing the blue and red curves from part (a), on a point-by-point basis, and represents the acoustic PSD (a.u.) received by the microphone (see main text).

In the absence of an external signal, the photodetector records the intrinsic, thermally driven vibrations of the buckled mirror. This yields the so-called thermomechanical noise spectrum (S_{WW}^{TM}, in units of W^2/Hz), plotted as the red solid line in Fig. S.2(a) for the sensor used here. The peaks at ~ 3.7 and ~ 6.6 MHz correspond to the lowest-order vibrational eigen-modes of the buckled mirror mechanical oscillator. As we have discussed extensively elsewhere [1-3], our sensors operate in a thermo-mechanical noise-limited regime wherein the shot-noise floor of the photodetector receiver lies well below S_{WW}^{TM} over a wide frequency range (extending above 10 MHz). External pressure waves induce additional motion of the cavity mirror, which can be treated as a linearly additive signal within limits set mainly by the tuned-to-slope readout technique [1].

As an example, the blue curve in Fig. S.2(a) is a typical spectrum recorded with the microphone positioned several mm above the piezoelectric transducer shown in the inset of Fig. S.1., with the transducer driven by the broadband 'noise source' setting of the function generator but with no additional object present. Thus, this spectrum represents the sum of the thermo-mechanical vibrations and the acoustic signal radiated by the transducer into the air above. Signal content, indicated by deviation from the background noise spectrum, is apparent over at least the ~ 0 to ~ 6 MHz range. The external signal can be isolated by dividing the measured spectrum on a point-by-point basis with the background noise spectrum [3]:

$$S_{PP}^{sig} = \left(\frac{S_{WW}^{tot} - S_{WW}^{TM}}{S_{WW}^{TM}}\right) NEP^2 \quad . \tag{S.1}$$

In this expression, S_{PP}^{sig} is the pressure spectral density (units of Pa^2/Hz) associated with the external acoustic signal, S_{WW}^{tot} is the total PSD due to the TM noise plus the external acoustic signal, and *NEP* is the noise-equivalent-pressure of the optomechanical sensor. This is valid provided that the thermomechanical noise is substantially higher than the background (shot) noise level [3]. A typical NEP for our sensors is on the order of ~ 50 – 100 $\mu Pa/Hz^{1/2}$ [1]. However, since the goal of our present study was to identify vibrational eigen-frequencies of an object rather than quantifying the received acoustic PSD, we simply set *NEP* to a value of unity in Eq. S.1, such that normalized plots of the acoustic PSD shown in the main manuscript are in arbitrary units (a.u.). In other words, we define S_{PP}^{sig} (a.u) ~ S_{WW}^{tot}/S_{WW}^{TM}, which furthermore assumes $S_{WW}^{tot} \gg S_{WW}^{TM}$ over a range of interest. For example, Fig. S.2(b) shows such a normalization procedure applied to the curves in part (a). This more clearly draws out the received acoustic energy attributable to the noise-driven transducer, with the intrinsic noise of the optomechanical sensor removed. The overall shape of this spectrum suggests a broadband response peaked near ~ 3 MHz. The 'ripple' in the spectrum (*i.e.*, the spectral positions of peaks and valleys) was highly dependent on the position of the microphone, both vertically and horizontally, relative to the piezoelectric transducer. We attribute this to acoustic interference effects, since the piezoelectric transducer itself has vibrational eigen-modes and thus nodes and anti-nodes associated with its surface displacement. Since our primarily goal was simply to identify vibrational eigen-frequencies of a subsequently placed object, we mostly neglected such secondary effects. In any case, the transducer used was sufficiently broadband to excite vibrational eigen-frequencies of a resting object over the ~ 0 – 6 MHz range and even up to ~ 10 MHz in some cases, as evidenced by the results in the main manuscript and below.

1.3. *Data collection and signal processing*

Acoustic signals created by the transducer-excited objects were sensed by the optomechanical microphone, and captured as 10 msec time-domain records by the digitizing photodetector receiver. A fast-Fourier-transform (FFT) algorithm (in MATLAB) was applied to the time trace to produce the frequency-domain spectra. For each measurement, 50 such records were captured and averaged. These averaged spectra were then gently smoothed using the 'smoothdata' function with the 'movmedian' preset with a 5-point window in MATLAB, and the signal spectrum was divided by the noise spectrum on a point-by-point basis, to produce the normalized, acoustic pressure spectral density (PSD) plots in arbitrary units (a.u) as discussed above.

2. Simulations

2.1. *Simulation Methods for Objects of Interest*

Different simulation methods were used depending on the material properties and geometries of the object of interest, as summarized in the following table.

Table S1. Table showing the material properties and simulation method used for each of the objects measured.

Object	Young's Modulus [GPa]	Poisson's Ratio	Density [g/cm³]	Simulation Method
1mm N-BK7 Ball Lens	82.0	0.206	2.51	COMSOL 2D-axisymmetric and COMSOL 3D
2mm N-BK7 Ball Lens	82.0	0.206	2.51	COMSOL 2D-axisymmetric
2mm Sapphire Ball Lens	N/A*	N/A*	3.98	Saviot piecewise-superquadric [4]
1.5mm Fused Silica Half Ball Lens	73.0	0.160	2.20	COMSOL 3D

*Anisotropic materials' Young's Modulus and Poisson's Ratio depend on the direction under which stresses are applied, therefore are described instead by an elasticity tensor. The elasticity tensor we used for sapphire is as follows [5]:

$$\begin{bmatrix} 496.8 & 163.6 & 110.9 & -23.5 & 0 & 0 \\ 163.6 & 496.8 & 110.9 & 23.5 & 0 & 0 \\ 110.9 & 110.9 & 498.1 & 0 & 0 & 0 \\ -23.5 & 23.5 & 0 & 147.4 & 0 & 0 \\ 0 & 0 & 0 & 0 & 147.4 & -23.5 \\ 0 & 0 & 0 & 0 & -23.5 & 166.6 \end{bmatrix} \text{[GPa]}$$

Each COMSOL simulation was performed using the "eigenfrequency" study under the solid mechanics physics toolbox. Simple isotropic spheres can be simulated by using the COMSOL 2D-axisymmetric simulation, which was performed for the perfectly spherical and isotropic 1mm and 2mm N-BK7 ball lenses. This simulation method solves for only spheroidal modes and does not predict degenerate and torsional modes. In short, a non-axisymmetric cross section of the object of interest is modeled, the material properties as given above are selected (which were already available in the COMSOL material library under Schott N-BK7 Glass), and then the eigenfrequency simulation takes place.

For objects with more sophisticated geometries and material properties such as the half ball and sapphire ball, COMSOL 2D-Axisymmetric simulations do not produce feasible results; thus, either a COMSOL 3D or Saviot piecewise-superquadric simulation [4] is required. After testing these methods out, Saviot piecewise-quadric simulations produced good agreement with experimental results for all objects measured, but COMSOL did not produce good fits in the case of the sapphire ball in particular. We were not able to identify the reason for this discrepancy, although it might arise due to numerical errors associated with mesh size and boundary conditions. Nevertheless, COMSOL was used for the other objects, particularly to produce images of the vibrational mode-field patterns, since the Saviot solver does not provide these. The COMSOL 3D solver produced good agreement with experimental results for the 1.5mm fused silica half ball and 1mm N-BK7 ball lens, but as mentioned not for the anisotropic sapphire ball lens. On the other hand, the Saviot on-line solvers [4] provided excellent fits for all objects studied. For all Saviot calculations, an N value of 12 was used. For all Saviot and COMSOL 3D simulations the spheroidal modes were identified and included in the figures in the main manuscript, whereas all purely torsional modes were ignored.

As stated in Table S1, we used a COMSOL 2D-Axisymmetric as well as COMSOL 3D simulation for the 1mm N-BK7 ball lens. The only reason we introduced the COMSOL 3D simulation was to properly simulate the slight anisotropy of the lens as described in section 3.2 in the main manuscript, because COMSOL 3D simulations properly simulate more sophisticated materials as stated above. Introducing anisotropy into the simulation was done by calculating the elasticity tensor of the perfectly isotropic Schott N-BK7 Glass material (which can be calculated from its Young's Modulus and Poisson's ratio), and slightly modifying the tensor's values until it fit the experimentally observed mode splitting. The original (isotropic) and modified (slightly anisotropic) elastic tensors used are as follows (in GPa):

$$\begin{bmatrix} 91.80 & 23.80 & 23.80 & 0 & 0 & 0 \\ 23.80 & 91.80 & 23.80 & 0 & 0 & 0 \\ 23.80 & 23.80 & 91.80 & 0 & 0 & 0 \\ 0 & 0 & 0 & 34.00 & 0 & 0 \\ 0 & 0 & 0 & 0 & 34.00 & 0 \\ 0 & 0 & 0 & 0 & 0 & 34.00 \end{bmatrix} \quad \begin{bmatrix} 91.39 & 23.85 & 23.85 & 0 & 0 & 0 \\ 23.85 & 91.39 & 23.85 & 0 & 0 & 0 \\ 23.85 & 23.85 & 91.39 & 0 & 0 & 0 \\ 0 & 0 & 0 & 34.43 & 0 & 0 \\ 0 & 0 & 0 & 0 & 34.43 & 0 \\ 0 & 0 & 0 & 0 & 0 & 34.43 \end{bmatrix}$$

(original) (modified)

2.2. *COMSOL Images of 2mm N-BK7 Ball Lens and 2mm Sapphire Ball Lens Modes*

As referenced in the main manuscript, COMSOL produces images of physical modes corresponding to each predicted eigenfrequency. Figure S.3(a) displays representative mode-field patterns for the 2mm N-BK7 ball lens, and Fig. S.3(b) shows them for the sapphire ball lens. The 2mm N-BK7 lens was simulated by performing a 2d-axisymmetric simulation; this simulation does not delineate the nominally degenerate modes or the purely torsional modes of the object. Thus, each photo in part (a) shows only one degenerate variation of each spheroidal mode. For the sapphire ball lens in part (b), simulated eigenfrequency values were obtained using Saviot's eigenfrequency calculator for piecewise-superquadratic objects [1]. These eigenfrequencies (as portrayed as the gray dotted lines in Figure 2(b) in the main manuscript) fit with experimental data much better than COMSOL's numerically predicted eigenfrequencies. That being said, the first 14 mode images in the figure below for the sapphire lens were predicted using a COMSOL 3D simulation (which includes degenerate modes), and were paired with the closest matching eigen-frequencies from Saviot. The sapphire case includes more than one mode image per eigen-frequency, exemplifying the degenerate modes for such an anisotropic object.

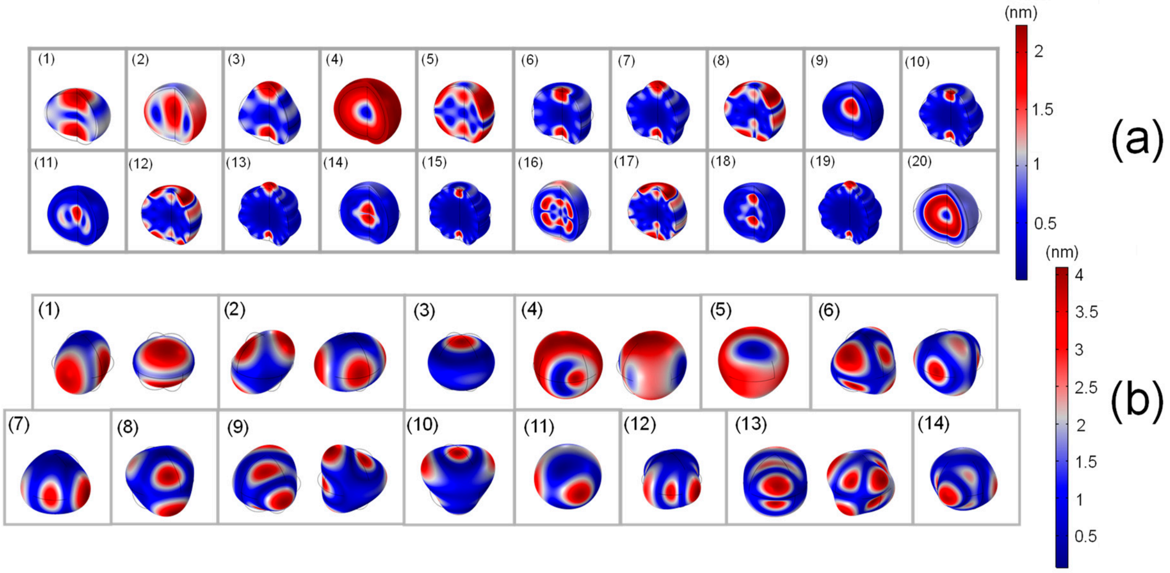

Figure S3. (a) Mode-field images for the 20 lowest-order spheroidal modes of a 2mm N-BK7 ball lens as simulated using COMSOL. (b) Mode-field images of the 14 lowest-order spheroidal modes (including degenerate modes) for the 2mm sapphire ball lens as simulated using COMSOL.

3. Supplementary Data

We also performed experiments on some other common objects, including ~ 2.5 mm diameter steel ball bearings and 1.59 x 3.17 mm dowel pins. For nominally isotropic chromium steel (a typical material for commonly manufactured ball bearings), scalar values for elastic modulus, density, and Poisson's coefficient were used and taken from the built-in COMSOL library [6]. COMSOL simulations were not performed on the stainless steel dowel due to the high density of modes within the frequency range of our measurement. Notably, our non-contact RUS technique with noise-based excitation of the piezo-electric transducer was able to yield high SNR spectra similar to those for the glass objects discussed in the main manuscript. Representative results are shown in Figure S.4. Compared to the glass and sapphire objects discussed above, steel has a higher acoustic impedance mismatch to air. Nevertheless, equally strong signatures of vibrational eigenmodes were detected by the optomechanical microphone. It should also be noted that the results in Fig. S.4 were obtained using a different transducer (than for all the other experiments discussed above). This transducer had a similar peak response frequency near ~ 3 MHz but a slightly smaller bandwidth, such that modes above ~ 5 MHz were not efficiently excited in the steel objects studied.

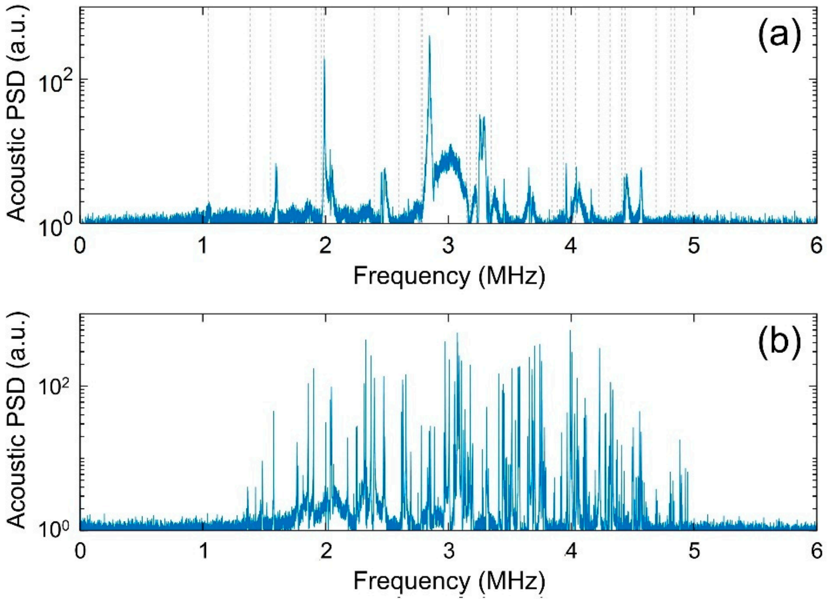

Figure S4. Observed normalized PSD vs. frequency spectra of: (a) a small steel bearing ball, and (b) a small steel dowel pin.

References

1. G. J. Hornig, K. G. Scheuer, E. B. Dew, R. Zemp, and R. G. DeCorby, "Ultrasound sensing at thermomechanical limits with optomechanical buckled-dome microcavities," Opt. Express **30**(18), 33083-33096 (2022).
2. K. G. Scheuer and R. G. DeCorby, "Air-coupled ultrasound using broadband shock waves from piezoelectric spark igniters," Appl. Phys. Lett. **125**, 082202 (2024).
3. K. G. Scheuer, A. Chen, G. Teves, and R. G. DeCorby, "Characterization of micro-scale gas leaks using an optomechanical ultrasound sensor," J. Acoust. Soc. Am., in press.
4. https://saviot.cnrs.fr/rus/tri/index.en.html, accessed June 25, 2025.
5. J. B. Wachtman, Jr., W. E. Tefft, D. G. Lam, and R. P. Stinchfield, "Elastic constants of synthetic single crystal corundum at room temperature," *J. Res. Natl. Inst. Stand. U.S. A.* 64A, 213–229 (1960)
6. https://www.comsol.com, accessed August 13, 2025.
